# Roxadustat in treating anemia in dialysis patients (ROAD): protocol and rationale of a multicenter prospective observational cohort study

**DOI:** 10.1186/s12882-021-02229-w

**Published:** 2021-01-13

**Authors:** Yaling Zhang, Song Ren, Hen Xue, Amanda Y Wang, Yang Zou, Yanrong Cai, Jingdong He, Xiaoling Yuan, Feifei Jiang, Jinxi Wei, Dongmei Yang, Dong He, Shide Hu, Min Lei, Fei Deng, Jin Chen, Xia Wang, Qiang He, Guisen Li, Daqing Hong

**Affiliations:** 1 Renal Department and Nephrology Institute, Sichuan Provincial People’s Hospital, School of Medicine, University of Electronic Science and Technology of China, No. 32, West 2nd Duan, 1st Circle Road, Qingyang District, Chengdu, Sichuan China; 2 Department of Nephrology, Ya’an People’s Hospital, 625000 Ya’an, Sichuan China; 3 The Renal and Metabolic Division, The George Institute for Global Health, University of New South Wales, Sydney, 2042 Australia; 4 Concord Clinical School, The University of Sydney, Sydney, 2042 Australia; 5 Department of Renal Medicine, Concord Repatriation General Hospital, Beijing Friendship Hospital, Beijing, China; 6 Department of Nephrology, 610000 Gao Xin Boli Hospital,Chengdu, China; 7 Department of Nephrology, The Second Affiliated Hospital of Chengdu Medical College, National Nuclear Corporation 416 Hospital, 610000 Chengdu, China; 8 Department of Nephrology, Sichuan Science City Hospital, 621000 Mianyang, China; 9 Department of Nephrology, Chengdu Jinniu District People’s Hospital, 610036 Chengdu, China; 10 Hemodialysis center,Pidu District People’s Hospital, 611730 Chengdu, China; 11 Department of Nephrology, Mianyang Anzhou People’s Hospital, 621000 Mianyang, China; 12 Department of Nephrology, The People’s Hospital of Mianyang, 621000 Mianyang, China; 13 Department of Nephrology, Sichuan Mianyang 404 Hospital, 621000 Mianyang, China; 14 Department of Nephrology, Affiliated Hospital of Chengdu University, 610081 Chengdu, China; 15 The George Institute for Global Health, Faculty of Medicine, University of New South Wales, Sydney, NSW Australia

**Keywords:** Roxadustat renal anemia hemodialysis

## Abstract

**Background:**

Roxadustat has been shown effective in treating patients with anemia due to chronic kidney disease. However, its long-term effect on clinical outcomes and socioeconomic burden and safety remains unclear.

**Methods/Design:**

This is a multicenter, prospective, longitudinal observational cohort study assessing if Roxadustat improves prognosis in dialysis patients. Primary outcomes will be major adverse cardiovascular events (MACE), defined as composites of cardiovascular death, myocardial infarction, cerebral infarction, hospitalization because of heart failure; all-cause mortality, and annual economic costs in two years. The data will be collected via Research electronic data capture (REDCap) based database as well as software-based dialysis registry of Sichuan province. The primary outcomes for the ROAD study participants will be compared with those in the dialysis registry cohort. Data at baseline and study follow up will also be compared to assess the association between Roxadustat and long-term clinical outcomes.

**Discussion:**

The main objective of this study is to the assess long-term association of Roxadustat on MACE, all-cause mortality, socio-economic burden, safety in dialysis patients, which will provide guidance for designing further large randomized controlled trials to investigate this clinic question.

**Study registration:**

The study has been registered in Chinese Clinical Trials Registry (ROAD, ROxadustat in treating Anemia in Dialysis patients, registration number ChiCTR1900025765) and provincial observational cohort database (Renal disEAse observational CoHort database, REACH, ChiCTR1900024926), registered 07 September 2019, http://www.chictr.org.cn.

## Background

The incidence and prevalence of Chronic kidney disease (CKD)is increasing globally which affects about 10% of people worldwide [1]. In China, the estimated number of people with CKD is 120 million [2], among which 2% will progress to end-stage renal disease (ESRD).

Renal anemia is one of the common complications of CKD especially ESRD, which is predominantly due to the reduction in erythropoietin (EPO) production by the kidney. Renal anemia can occur in any stages of CKD, but its prevalence and severity increases with the progression of CKD [3]. It is reported that more than 90% of dialysis patients have renal anemia [4], which is associated with increased risk of cardiovascular events [5, 6] and all-cause mortality [7].

Erythropoiesis-stimulating agents (ESA) are commonly used in the treatment of renal anemia. It can not only improve hemoglobin levels, but also cardiovascular outcomes in CKD patients. It can also delay the progression of chronic kidney disease, reduce hospitalization, all-cause mortality, and improve the quality of life of CKD patients [8]. The DOPPS study found that for every 10 g/L increase of hemoglobin (HGB), the risk of death and hospitalization decreased by 5% and 4% respectively [9]. However, the use of ESA also carries adverse events. For example, high dose of ESA is associated with increased risk of cardiovascular events and mortality, and regardless of HGB levels [10], demonstrated by the CHOICE study. In addition, ESA resistance can occur in some patients, resulting in treatment failure.

In recent years, hypoxia inducible factor (HIF) related medications have been tested in the treatment of renal anemia in a number of trials. HIF can increase the production of endogenous EPO by inhibiting the activity of prolyl hydroxylase. Roxadustat, as a HIF prolyl hydroxylase inhibitor (HIF-PHI), has been completed Clinical trials in several countries. The most recent randomized, multicenter, double-blind clinical studies, done in China, have found that Roxadustat significantly improved the hemoglobin level in CKD patients on and not on dialysis [8, 11]. Other studies also showed Roxadustat can effectively correct renal anemia, and reduce the requirements for blood transfusion and intravenous iron supplementation [3, 12, 13].

Despite the proven short-term benefits of Roxadustat in CKD patients, its long-term effect on clinical outcomes and socio-economic burden remains uncertain. Therefore, this study aims to perform a prospective longitudinal cohort study assessing the long-term effect of Roxadustat on cardiovascular events and mortality, as well as economic costs in dialysis patients with renal anemia.

## Methods/Design

### Study design

This is a multicenter, prospective, longitudinal cohort study. It has been registered in Chinese Clinical Trials Registry (ROAD, ROxadustat in treating Anemia in Dialysis patients, registration number ChiCTR1900025765), provincial observational cohort database (Renal disEAse observational CoHort database, REACH, ChiCTR1900024926).

### Study population

The participants included in the ROAD study will be patients with renal anemia in the context of end-stage kidney disease on dialysis (both hemodialysis and peritoneal dialysis), and on Roxadustat treatment who consent to the study. The de-identified data from the provincial dialysis registry of dialysis patients with renal anemia will be serves as a control group. The study was approved by the Institutional Review Board at Sichuan Provincial People’s Hospital (2019 − 196). The informed consent obtained from study participants will be written.

Inclusion criteria include:①Age 18–90 years; ②End-stage kidney disease patients who are receiving maintenance hemodialysis or peritoneal dialysis;③Dry weight 45–160 kg; ④Renal anemia, defined as HGB < 130 g/(male), HGB < 120 g/L(female) or HGB is maintained normal by ESA; ⑤willing to participate in the study with informed consent. Exclusion criteria includes:①patients with pre-existing malignancy; ②Known history of hematological disorders including myelodysplastic syndrome, multiple myeloma, hereditary hematologic disease such as thalassemia, sickle cell anemia, pure red cell aplasia, or other known causes for anemia other than CKD or dialysis. ③Patients who cannot complete the follow-up study.

### Study outcomes

Primary outcomes will be major adverse cardiovascular events (MACE), defined as composite of cardiovascular death, myocardial infarction, cerebral infarction, hospitalization because of heart failure or unstable angina pectoris; all-cause mortality and annually medical economic costs in 2 years.

Secondary outcomes will include: ①Mean HGB changes from baseline to the 16th week and percentage of patients within HGB treatment target (110–130 g/L). ②Changes from baseline and variations in the mean HGB level throughout the whole study periods (up to 108 weeks). ③Changes of serum iron metabolism indexes (Hepcidin, ferritin, transferrin, transferrin saturation, and total iron binding capacity) as well as requirements for intravenous iron replacement from baseline to the end of the study(up to 108 weeks). ④Changes of blood pressure and antihypertensive medications from baseline to 16th week. ⑤Changes of serum lipids profile from baseline to 16th week. ⑥Changes of the following laboratory results (collected in the Sichuan provincial dialysis registration system) from baseline to the end of study, dialysis related parameters, dialysis adequacy and chronic kidney disease- mineral bone disorder (CKD-MBD) markers (serum phosphorus, calcium, and parathyroid hormone). ⑦Mean weekly dosage of Roxadustat to maintain HGB within the treatment target. ⑧Adverse events (AE) during the follow-up period, with a focus on drug discontinuation due to AE. ⑨Annual economic cost related to hospitalization, outpatient treatments, medical insurance coverage and out-of-pocket payments.

### Data collection

The de-identified data were collected in REACH via Research electronic data capture (REDCap [14, 15], hosted at Sichuan Provincial People’s Hospital) pre-designed forms from baseline to week 108/2 years. The table with an identification code will be kept within the study site in a secure way. The provision of de-identified medical records will be required for judication for the MACE outcome. Participants will be followed up to 2 years except that they withdraw from the study, have their dialysis type changed, receive kidney transplantation or have lost follow up.

Dialysis data from each study site in the provincial dialysis registry will be collected via a dialysis software provided by the Sichuan Quality Control Center of Renal Disease at the end of the first and second study year.

A questionnaire focusing on medical expense for the participants will be collected at the end of the first and second year of the study.

The target sample size of the ROAD participant is greater than 250 basing on the MACE incidence of at least 20% [16, 17] within 2 years among ROAD and dialysis registry cohort, basing on noninferiority assumption with a marginal difference of 0.02 (10% of MACE incidence), actual difference of -0.08 (40% of MACE incidence),α = 0.05 and 1-β = 0.8, allowing a drop rate of 15%, using ‘Non-Inferiority Tests for the Difference Between Two Proportions’ of PASS software 15.0.5.

### Quality control

The investigators will be trained before initiation of the study. A multi-disciplinary team will provide consultation on adjustment of Roxadustat dosage based on HGB level if needed during the study. An online video and handbook will be provided to guide the data entry. The online forms will be provided for data validation to minimized errors with data entry. Data monitoring will be carried out to make sure the accuracy of the data entry. The main outcome of MACE will be judicated independently by two trained doctors using de-identified medical records. A third doctor will be consulted if there are any disagreements. The questionnaire on economic costs will be randomly selected and double-checked with medical insurance department to ensure the authenticity. The data will be locked once the data cleaning completes, and the analysis plan will be prepared and run independently by two statisticians.

### Data analysis

The comparison in primary outcomes between the ROAD cohort and the registry data will be performed using Multiple Cox regression of risk factors and propensity score method will also be applied as a sensitivity analysis. The medical economic costs will be compared between ROAD cohort and registry control group using the whole population as well as populations stratified by hospital levels, regions, comorbidities.

The efficacy on HGB of Roxadustat in the treatment of anemia of the whole cohort as well as the pre-defined subgroups (including patients with evidence of erythropoietin resistance, patients with chronic inflammation, patients with impaired iron metabolism, and patients on hemodialysis or peritoneal dialysis) will be analyzed in intention-to-treat and per-protocol ROAD population. Treatment heterogeneity will be tested by adding an interaction term to the statistical models.

## Discussion

Anemia is a common complication in patients with chronic kidney disease, especially in those on dialysis. Treatment of renal anemia is associated with improvement in cardiovascular function, and health related quality of life, reducing requirements for blood transfusion and hospitalization [18–20]. Erythropoiesis-stimulating agents are one of main treatments for anemia in dialysis [21]. Higher dosage of erythropoiesis-stimulating agents, however, is associated with risk of stroke [22–24], however, potential risks may occur according [25–28]. In addition, more than 10% patients were hyporesponsive to ESA [29, 30].

Roxadustat, a potent, reversible, HIF-PHI that mimics the natural response to hypoxia results in target genes involved in erythropoiesis [31–33]. The meta-analysis of randomized controlled trials showed that Roxadustat can improve hemoglobin levels and iron metabolism in both dialysis-dependent and non-dialysis-dependent chronic kidney disease [34, 35]. However, its long term efficacy and safety needs to be investigated.

This study is designed to systemically assess the impact of Roxadustat on dialysis patients in the real-world practice. The sites were chosen from different levels and different regions in the Sichuan province to make sure the repetitiveness of the study population. All treatment decision is made by the treating physician according to the patient’s clinical conditions; however, the multi-disciplinary team will provide consultation if required to improve the treatment of anemia. The data is collected both manually input and using software-based registry, which could increase the efficiency and reduce the typing errors. The software will be informative to assist doctors in treating dialysis patients with drug information, clinical guidelines and important alerts.

The main objective of the study is to provide the long-term impact of Roxadustat on MACE and all-cause mortality, economic burden, and safety. Differences in responding to Roxadustat in different subgroups will also be assessed. This study will be able to provide guidance in designing future, large scale randomized controlled trials assessing the impact of Roxadustat in dialysis patients.

## Data Availability

Not applicable at this stage. The datasets analyzed during the current study will be available from the corresponding author on reasonable request when the study is finished.

## Abbreviations

REDCap: Research electronic data capture
ROAD: Roxadustat in treating anemia in dialysis patients
REACH: Renal disEAse observational CoHort database
CKD: Chronic kidney disease
ESRD: End-stage renal disease
EPO: Erythropoietin
ESA: Erythropoietin-stimulating agents
the DOPPS study: The Dialysis Outcomes and Practice Patterns Study
HGB: Hemoglobin
the CHOICE study: The Choices for Healthy Outcomes in Caring for ESRD study
HIF: Hypoxia inducible factor
HIF-PHI: Hypoxia inducible factor-prolyl hydroxylase inhibitor
MACE: Major adverse cardiovascular events
CKD-MBD: Chronic kidney disease-mineral bone disorder
AE: Adverse events
